# Demonstration of the Relationship between Sociodemographic Characteristics and Depression in Patients with Carpal Tunnel Syndrome

**DOI:** 10.1055/s-0045-1806825

**Published:** 2025-06-14

**Authors:** Şule Göktürk, Yasin Göktürk, Belgin Oral, Eda Başmısırlı

**Affiliations:** 1 Department of Neurosurgery, Kayseri City Hospital, University of Health Sciences, Kayseri, Republic of Türkiye.; 2 Department of Public Health, Occupational Disease Clinic, Kayseri City Hospital, University of Health Sciences, Kayseri, Republic of Türkiye.; 3 Department of Nutrition and Dietetics, Faculty of Health Sciences, Nuh Naci Yazgan University, Kayseri, Republic of Türkiye.

**Keywords:** anxiety, carpal tunnel syndrome, depression, ansiedade, depressão, síndrome do túnel do carpo

## Abstract

**Objective:**

To present the clinical, epidemiological, and socioeconomic characteristics of individuals with symptoms of carpal tunnel syndrome (CTS) and depression.

**Methods:**

The Beck Depression Inventory (BDI) was administered to 100 patients diagnosed with CTS, and its relationship between anxiety and depression was investigated alongside the sociodemographic characteristics of the patients.

**Results:**

Depression was identified in 30.9% of the 68 patients, the majority of whom were women. The BDI scores were found to be significantly higher in patients with low socioeconomic status, poor nutrition, and smoking ( *p* < 0.05). Furthermore, the frequency of depressive symptoms was found to be significantly higher in patients who had chronic low back and neck pain along with CTS, with regular use of analgesics, and who had previously undergone any surgery ( *p* < 0.05).

**Conclusion:**

Depression and anxiety are prevalent among individuals diagnosed with CTS. Effective treatment, including the management of comorbid depression and anxiety, is crucial for optimal patient outcomes.

## Resumo

**Objetivo:**

Apresentar as características clínicas, epidemiológicas e socioeconômicas de indivíduos com sintomas de síndrome do túnel do carpo (STC) e depressão.

**Métodos:**

O Inventário de Depressão de Beck (IDB) foi administrado a 100 pacientes diagnosticados com STC e sua relação entre ansiedade e depressão foi investigada juntamente com as características sociodemográficas dos pacientes.

**Resultados:**

A depressão foi identificada em 30,9% dos 68 pacientes, em sua maioria mulheres. As pontuações no IDB foram significativamente maiores em pacientes com condição socioeconômica baixa, má nutrição e tabagismo ( *p* < 0,05). Além disso, a frequência de sintomas depressivos foi significativamente maior em pacientes com dor lombar e cervical crônica além da STC, com uso regular de analgésicos e que já haviam sido submetidos a alguma cirurgia ( *p* < 0,05).

**Conclusão:**

Depressão e ansiedade são prevalentes entre indivíduos diagnosticados com STC. O tratamento eficaz, incluindo o controle da depressão e ansiedade comórbidas, é crucial para bons desfechos.

## Introduction

Carpal tunnel syndrome (CTS) is a condition that arises as a consequence of compression of the median nerve at various levels within the carpal tunnel at the wrist. It represents the most prevalent peripheral neuropathy in the upper extremity. 1 Despite the lack of clarity surrounding its pathogenesis, it is observed with greater frequency in the middle-aged and women. It is also possible for occupational CTS to occur in individuals who are subjected to pressure on the flexor retinaculum, located on the inner aspect of the hand. However, it is important to note that the prevalence of this condition is also high in individuals with obesity, pregnancy, diabetes, chronic alcohol use, and rheumatological diseases.

Occupational CTS is a rare phenomenon, and it is essential to exclude all other potential causes, particularly factors such as obesity, before attributing the condition to occupational factors. The most prevalent mechanisms in the pathogenesis of this condition are mechanical compression, microvascular insufficiency and vibration theories. The mechanical compression theory posits that the symptoms of CTS are caused by compression of the median nerve within the carpal tunnel. 1

The prevalence of CTS and its distribution in society can vary considerably, with estimates ranging from 0.125 to 1% and 5 to 16%. The etiology or underlying cause is also a significant factor in determining its incidence. 2 The two principal forms of CTS are acute and chronic. The former is less common and arises when the pressure within the carpal tunnel increases rapidly. The condition was first described by Sir James Paget in 1854, in the context of a fractured radius. 3 In their study, Silverstein et al. investigated the correlation between high-force/repetitive movements and CTS among a cohort of 652 workers across 39 occupations, in 7 distinct industrial sectors. Their findings indicated that repetitive movements may represent a significant risk factor for CTS. However, they also suggested that high force and repetition alone may not be sufficient to induce the condition. 4

The classic symptoms of CTS include paresthesia, numbness and tingling in the areas innervated by the median nerve, particularly at night. Several tests are employed in the diagnosis of CTS, including the Tinel, Phalen, square wrist, closed fist, and flick signs, as well as the Katz hand diagram, the wrist flexion and extension test, the pressure provocation test, and the tourniquet test.

The symptoms may vary depending on the severity of the disease. During the initial phase, symptoms manifest as a result of the involvement of the median nerve's sensory component. In contrast, during the subsequent phase, symptoms emerge as a consequence of the involvement of motor fibers. The most frequently observed symptom is burning pain accompanied by paresthesia and numbness in the distribution of the median nerve in the distal wrist. The affected fingers are the thumb, index finger and middle finger, and the radial half of the ring finger. Patients frequently report nocturnal pain and paresthesia, with attempts to alleviate their symptoms by squeezing their hands or changing positions. The diagnosis can be established through the use of electromyography (EMG), ultrasound (US), and magnetic resonance imaging (MRI). 5

A variety of surgical and nonsurgical treatment options are available. Nonsurgical methods are effective in patients with mild to moderate CTS. Corticosteroid injections into the wrist is a frequently employed method by physical therapy or algology physicians. Although symptoms may temporarily worsen, the treatment can provide complete or significant pain relief for weeks to years in 60 to 70% of patients. 6 7 8 In surgical procedures, the transverse carpal ligament is incised and the nerve is relaxed, thereby performing a decompression operation.

Individuals diagnosed with CTS may perceive their condition as a barrier to continued employment, which could potentially lead to financial challenges. Additionally, they may experience physical discomfort and a sense of diminished strength, which could contribute to a range of emotional and psychological difficulties. These may include depressive symptoms such as anxiety, reduced interest and engagement in daily activities, as well as feelings of dissatisfaction and sadness. It has been documented that individuals afflicted with physical ailments are more prone to experiencing feelings of anxiety and depression. 9 Also, it has been demonstrated that mental health conditions, such as depression and anxiety, can result in functional limitations among patients with upper extremity musculoskeletal disorders. 10

The objective of this study is to examine the relationship between anxiety and depression status and the disease in CTS patients using the Beck Depression Inventory (BDI).

## Materials and Methods

This study was conducted by the hospital's Neurosurgery Clinic between March and June 2024. Those presenting to the outpatient clinic with CTS were first informed about the study and their written consent was obtained, in accordance with the principles of the Declaration of Helsinki. The study was approved by our hospital's Ethical Committee under the number 76397871, dated March 15 ^th^ , 2024.

### Study Design

The sample of the study was obtained as 100, using literature information and the Number Cruncher Statistical System (NCSS) 2007 and Power Analysis and Sample Size (PASS) 2008 statistical softwares (NCSS LLC., Kaysville, UT, USA). The study population comprised 100 CTS patients aged between 18 and 72 years, including those with mild, moderate and severe disease, irrespective of gender. A questionnaire was administered to the patients, comprising 21 questions. These enquired about sociodemographic data, including age, gender, place of residence, schooling, and economic status. Four of the questions were open-ended and focused on behavior regarding smoking and alcohol use, exercise and nutrition. Additionally, we included the 21-question Beck depression Inventory (BDI). The data were collected by a single researcher using the face-to-face interview method. Patients' height (in centimeters), body weight (in kilograms), and body mass index (BMI) were calculated on a weighing and measuring scale with 0.01 kg precision, with the subject barefoot and wearing sports clothes.

### Exclusion Criteria

The study's population comprises individuals with neurological conditions affecting the central nervous system, resulting in neurological deficits in the upper extremity. Additionally, the cohort includes individuals diagnosed with polyneuropathy.

### Beck Depression Inventory

The BDI is frequently employed in our country. It comprises 21 questions, is a survey instrument with four response options, yielding scores between 0 and 3. The Turkish validity and reliability of the BDI were established by Hisli. 11

### Statistical Analysis

The data were recorded using the IBM SPSS Statistics for Windows (IBM Corp., Armonk, NY, USA) software, version 22.0, and the analyses were performed using the same program. Descriptive statistics included frequency, percentage, mean, standard deviation (SD), median, and range values. To conduct a statistical analysis of categorical data, a Yates correction was applied to values below 25, and a Fisher's exact test was employed for values below 5. The Kolmogorov-Smirnov test was employed to ascertain whether the data exhibited a normal distribution. In binary groups where the quantitative data did not conform to a normal distribution in independent groups, the Mann-Whitney U-test was utilized. For groups comprising more than two categories, the Kruskall-Wallis test (post hoc Dunn's) was applied. The statistical significance of the observed difference was deemed to be *p* < 0.05.

## Results

The mean age of the 100 CTS patients who participated in the study was 44.76 ± 13.45 (range: 18–72) years, with 68% of the cohort being female. The mean BDI score of the patients was 11.99 ± 7.42 (range: 0–34). When the cut-off point was taken as 17 and above, the frequency of depressive symptoms was found to be 26%. While BDI scores were relatively low among university graduates, they were significantly higher among those who defined their economic situation as poor, residing in rural areas, and unemployed ( *p* < 0.05), as shown in Table 1 . In our study, the frequency of depressive symptoms was significantly higher in CTS patients who described their diet as irregular, did not engage in regular physical activity, had previously received psychological help, and had previous antidepressant use ( *p* < 0.05). Furthermore, BDI scores were found to be significantly lower in smokers and alcohol users ( *p* < 0.05). However, the observed difference was not statistically significant ( *p* > 0.05), as shown in Table 2 .

**Table 1:** Sociodemographic findings and BDI scores of CTS patients

Variables		n	BDI score			*p* -value	Depression frequency		*p* -value
			Median	Range	Mean ± SD		Positive: n (%)	Negative: n (%)	
**Gender**	Female	68	10.5	0–37	12.0 ± 7.3	0.827*	21 (30.9)	47 (69.1)	0.168***
	Male	32	11.0	0–34	11.9 ± 7.3		5 (15.6)	27 (84.4)	
**Age (years)**	≤ 40	42	11.0	0–26	11.4 ± 6.5	0.622*	8 (19.0)	34 (81.0)	0.264***
	> 40	58	10.0	0–34	12.4 ± 8.0		18 (31.0)	40 (69.0)	
**Schooling**	Under high school	66	12.5	0–34	12.8 ± 7.8	0.029**	20 (30.3)	46 (69.7)	0.144****
	High school	25	11.0	3–26	11.8 ± 6.5		6 (24.0)	19 (76.0)	
	University	9	6.0	0–13	6.1 ± 4.0		0 (0.0)	9 (100.0)	
**Economic status**	Good	35	8.0	0–27	10.5 ± 7.2	0.032**	6 (17.1)	29 (82.9)	0.037****
	Medium	61	11.0	0–29	12.2 ± 7.1		17 (27.9)	44 (72.1)	
	Bad	4	20.5	13–34	22.0 ± 8.7		3 (75.0)	1 (25.0)	
**Family type**	Small	69	10.0	0–34	11.8 ± 7.8	0.488*	17 (24.6)	52 (75.4)	0.828***
	Big	31	13.0	0–26	12.5 ± 6.7		9 (29.0)	22 (71.0)	
**Habitation**	City	68	9.0	0–34	10.9 ± 7.3	0.013*	13 (19.1)	55 (80.9)	0.041***
	Town	32	14.5	0–27	14.4 ± 7.2		13 (40.6)	19 (59.4)	
**Working Status**	Yes	39	8.0	0–26	9.2 ± 6.3	0.001*	4 (10.3)	35 (89.7)	0.008***
	No	61	13.0	0–34	13.8 ± 7.6		22 (36.1)	39 (63.9)	

**Abbreviations** : BDI, Beck Depression Inventory; CTS, carpal tunnel syndrome; SD, standard deviation.

**Notes** : *Mann-Whitney U-test. **Kruskal-Wallis test. ***Chi-squared Yates correction. ****Fisher's exact test.

**Table 2:** Health and behavioral status and BDI scores in CTS patients

Variables		n	BDI score			*p* -value	Depression frequency		*p* -value
			Median	Range	Mean ± SD		Positive: n (%)	Negative: n (%)	
**Chronic disease**	Yes	**44**	**11.5**	**0–34**	**12.9 ± 8.5**	0.470*	**15 (34.1)**	**29 (65.9)**	0.160***
	No	56	10.0	0–26	11.3 ± 6.4		11 (19.6)	45 (80.4)	
**Regular medication**	In use	42	12.0	0–29	12.9 ± 7.9	0.314*	15 (35.7)	27 (64.3)	0.098***
	No	58	10.0	0–34	11.3 ± 7.0		11 (19.0)	47 (81.0)	
**Chronic disease in family members**	Var	28	11.0	0–29	11.3 ± 7.4	0.612*	6 (21.4)	22 (78.6)	0.692***
	Yok	72	10.5	0–34	12.3 ± 7.5		20 (27.8)	52 (72.2)	
**Nutritional status**	Regular	74	10.0	0–27	11.1 ± 6.7	0.105*	15 (20.3)	59 (79.7)	0.028***
	Irregular	26	13.0	0–34	14.4 ± 8.8		11 (42.3)	15 (57.7)	
**Regular Exercise**	Yes	22	9.0	3–24	9.7 ± 5.5	0.122*	2 (9.1)	20 (90.9)	0.041***
	No	78	12.0	0–34	12.6 ± 7.8		24 (30.8)	54 (69.2)	
**Tobacco use**	In use	38	8.5	0–34	10.4 ± 7.8	0.049*	7 (18.4)	31 (81.6)	0.264***
	No	62	13.0	0–29	12.9 ± 7.1		19 (30.6)	43 (69.4)	
**Alcoholism**	In use	12	8.5	0–34	9.6 ± 6.2	0.203*	1 (8.3)	11 (91.7)	0.177****
	No	88	12.0	3–26	12.3 ± 7.5		25 (28.4)	63 (71.6)	
**Previous psychological treatment**	Yes	9	18.0	5–27	16.4 ± 8.1	0.082*	5 (55.6)	4 (44.4)	0.049****
	No	91	10.0	0–34	11.5 ± 7.3		21 (23.1)	70 (76.9)	
**Previous antidepressant use**	Yes	9	23.0	8–27	19.3 ± 7.0	0.003*	6 (66.7)	3 (33.3)	0.009****
	No	91	10.0	0–34	11.3 ± 7.1		20 (22.0)	71 (78.0)	
** BMI (kg/m ^2^ ) **	< 25	16	12.0	0–34	12.9 ± 9.4	0.148**	3 (18.8)	13 (81.3)	0.123
	25–29.9	44	9.0	0–24	10.3 ± 6.6		8 (18.2)	36 (81.8)	
	≥ 30	40	13.0	0–29	13.4 ± 7.3		15 (37.5)	25 (62.5)	

**Abbreviations** : BDI, Beck Depression Inventory; BMI, body mass index; CTS, carpal tunnel syndrome; SD, standard deviation.

**Notes** : *Mann-Whitney U-test. **Kruskal-Wallis test. ***Chi squared Yates correction. ****Fisher's exact test.

The prevalence of depressive symptoms was significantly higher in patients with CTS who had chronic low back and neck pain, used regular analgesics for this pain, and had previously undergone any surgery ( *p* < 0.05), as shown in Table 3 .

**Table 3:** Chronic low back-neck pain and BDI scores in CTS patients

Variables		n	BDI score			*p* -value	Depression frequency		*p* -value
			Median	Range	Mean ± SD		Positive: n (%)	Negative: n (%)	
**Chronic neck pain**	Yes	47	13.0	0–34	14.2 ± 8.2	0.009*	21 (44.7)	26 (55.3)	< 0.001**
	No	53	9.0	0–29	10.0 ± 6.1		5 (9.4)	48 (90.6)	
**Chronic back pain**	Yes	41	11.0	0–34	13.4 ± 8.7	0.232*	17 (41.5)	24 (58.5)	0.007**
	No	59	11.0	0–29	11.0 ± 6.3		9 (15.3)	50 (84.7)	
**Analgesic for low back and neck pain**	Yes	50	12.5	0–27	13.1 ± 7.2	0.102*	18 (36.0)	32 (64.0)	0.040**
	No	50	9.5	0–34	10.8 ± 7.5		8 (16.0)	42 (84.0)	
**Previous surgery**	Yes	49	13.0	0–34	13.8 ± 8.2	0.029*	18 (36.7)	31 (63.3)	0.030**
	No	51	9.0	0–24	10.2 ± 6.2		8 (15.7)	43 (84.3)	

**Abbreviations** : BDI, Beck Depression Inventory; CTS, carpal tunnel syndrome; SD, standard deviation.

**Notes** : *Mann-Whitney U-test.**Fisher's exact test.

## Discussion

The present study revealed that the prevalence of depressive symptoms was 26%. Notably, BDI scores were observed to be relatively low among university graduates. However, they were found to be significantly elevated among individuals who self-identified as economically disadvantaged, rural area residents, and unemployed.

In patients with CTS, pain, paresthesia and tingling sensations in the area innervated by the nerve have a detrimental impact on quality of life. This condition has a profound impact on physical function in individuals with persistent hypoesthesia and motor impairment. It can also give rise to mental health problems, such as anxiety or depression. 12 McCallum et al. showed a link between anxiety and depression levels and the presence of pain in their study. 13 This is corroborated by prior research examining the incidence of mental health illnesses in individuals with chronic pain. 14 Additionally, it has been demonstrated that social circumstances and physical activities that influence health may be linked to mental health in many musculoskeletal and neurological disorders. 15 16

In accordance with the findings of Adjibade et al., 17 patients who exhibited multiple healthy lifestyle behaviors (i.e., nonsmoking, low alcohol intake, regular physical activity, a healthy diet, and a normal BMI) demonstrated a reduced likelihood of experiencing depressive symptoms in comparison to those with two or fewer healthy lifestyle behaviors. In our study, the frequency of depressive symptoms was significantly higher in subjects who described their diet as irregular, did not engage in regular physical activity, had previously received psychological help, and had previously used antidepressants. Additionally, BDI scores were significantly lower in smokers.

In a study conducted in 2020, Paiva Filho et al. 18 evaluated the prevalence of anxiety and depression in CTS patients. Although no association was found between BMI and higher depression or anxiety index when considered alone, almost ⅔ of the patients exhibited values above the normal range. In their study, the characteristics that were statistically associated with both anxiety and depression symptoms in patients with CTS, independent of other characteristics, were gender, smoking status, and family income. In our study, lower BDI values were also correlated with alcohol intake, but the difference was not statistically significant.

The relationship between depressive symptoms and surgical outcomes in CTS is of particular interest, given the high prevalence of both conditions, particularly in women. 19 In the present study, depression was identified in 30.9% of the 68 female patients. The BDI scores were relatively low among university graduates, but significantly higher among those who described their economic situation as poor, who resided outside the city (in towns and villages), and who were unemployed. Once more, the existing literature indicates a statistically significant association between smoking and family income and anxiety in patients with CTS. 19 Depression alone was found to have a statistically significantly association with family income in CTS patients. Studies have shown that conditions such as depression and anxiety may contribute to functional limitations in patients with upper extremity musculoskeletal disorders. 20

Furthermore, our study demonstrated that the prevalence of depressive symptoms was markedly elevated in patients presenting with chronic lower back and neck pain in conjunction with CTS, with regular analgesic use, and who underwent prior surgical procedures ( *p* < 0.05).

It is of great importance to gain an understanding of the sociodemographic characteristics of CTS individuals with mood disorders, particularly anxiety and depression, to provide optimal treatment and facilitate healing following surgical intervention. The present study is informed by a small number of articles published on similar topics, as well as by the broader literature on the subject. The aim was to examine the clinical, epidemiological and socioeconomic characteristics of individuals presenting with CTS, anxiety, and depression symptoms.

## Conclusion

People with CTS had a high prevalence of symptoms of depression and anxiety. Treatment is of utmost importance for the recovery of patients. More research is needed aimed at demonstrating the effectiveness of approaches that address organic and pathological causes, as well as psychosocial stressors, anxiety, and depression, on relieving CTS symptoms.
